# Long-term Effectiveness of a Multistrategy Behavioral Intervention to Increase the Nutritional Quality of Primary School Students’ Online Lunch Orders: 18-Month Follow-up of the Click & Crunch Cluster Randomized Controlled Trial

**DOI:** 10.2196/31734

**Published:** 2021-11-29

**Authors:** Rebecca Wyse, Tessa Delaney, Fiona Stacey, Christophe Lecathelinais, Kylie Ball, Rachel Zoetemeyer, Hannah Lamont, Rachel Sutherland, Nicole Nathan, John H Wiggers, Luke Wolfenden

**Affiliations:** 1 Hunter New England Population Health Wallsend Australia; 2 School of Medicine and Public Health University of Newcastle Callaghan Australia; 3 Priority Research Centre for Health Behaviour University of Newcastle Callaghan Australia; 4 Hunter Medical Research Institute New Lambton Australia; 5 Institute for Physical Activity and Nutrition Deakin University Melbourne Australia

**Keywords:** child diet, consumer behavior, intervention, RCT, public health nutrition, obesity, school, school canteen, long-term follow-up, choice architecture, public health, nutrition, children, diet, eHealth, school lunch

## Abstract

**Background:**

School food services, including cafeterias and canteens, are an ideal setting in which to improve child nutrition. Online canteen ordering systems are increasingly common and provide unique opportunities to deliver choice architecture strategies to nudge users to select healthier items. Despite evidence of short-term effectiveness, there is little evidence regarding the long-term effectiveness of choice architecture interventions, particularly those delivered online.

**Objective:**

This study determined the long-term effectiveness of a multistrategy behavioral intervention (Click & Crunch) embedded within an existing online school lunch-ordering system on the energy, saturated fat, sugar, and sodium content of primary school students’ lunch orders 18 months after baseline.

**Methods:**

This cluster randomized controlled trial (RCT) involved a cohort of 2207 students (aged 5-12 years) from 17 schools in New South Wales, Australia. Schools were randomized to receive either a multistrategy behavioral intervention or the control (usual online ordering only). The intervention strategies ran continuously for 14-16.5 months until the end of follow-up data collection. Trial primary outcomes (ie, mean total energy, saturated fat, sugar and sodium content of student online lunch orders) and secondary outcomes (ie, the proportion of online lunch order items that were categorized as *everyday*, *occasional*, and *caution*) were assessed over an 8-week period at baseline and 18-month follow-up.

**Results:**

In all, 16 schools (94%) participated in the 18-month follow-up. Over time, from baseline to follow-up, relative to control orders, intervention orders had significantly lower energy (–74.1 kJ; 95% CI [–124.7, –23.4]; *P*=.006) and saturated fat (–0.4 g; 95% CI [–0.7, –0.1]; *P*=.003) but no significant differences in sugar or sodium content. Relative to control schools, the odds of purchasing *everyday* items increased significantly (odds ratio [OR] 1.2; 95% CI [1.1, 1.4]; *P*=.009, corresponding to a +3.8% change) and the odds of purchasing *caution* items significantly decreased among intervention schools (OR 0.7, 95% CI [0.6, 0.9]; *P*=.002, corresponding to a –2.6% change). There was no between-group difference over time in canteen revenue.

**Conclusions:**

This is the first study to investigate the *sustained* effect of a choice architecture intervention delivered via an online canteen ordering systems in schools. The findings suggest that there are intervention effects up to 18-months postbaseline in terms of decreased energy and saturated fat content and changes in the relative proportions of healthy and unhealthy food purchased for student lunches. As such, this intervention approach may hold promise as a population health behavior change strategy within schools and may have implications for the use of online food-ordering systems more generally; however, more research is required.

**Trial Registration:**

Australian New Zealand Clinical Trials Registry ACTRN12618000855224; https://www.anzctr.org.au/Trial/Registration/TrialReview.aspx?id=375075

## Introduction

Poor diet is a leading cause of death and disability globally [1]. As dietary patterns in childhood track through to adulthood and are predictive of future disease [2], improving child nutrition is a global public health priority [3]. School food services, including cafeterias and canteens, are an ideal setting in which to improve child nutrition [4]. In Australia, school canteens are accessed by up to 95% of Australian children [5,6] to buy food and drinks during meal and snack times. Energy-dense, nutrient-poor foods and foods high in saturated fat, salt, or sugar are frequently available at school canteens and are commonly purchased by students [1,7-9]. As such, interventions to improve the nutritional quality of foods purchased at school canteens are warranted.

Choice architecture interventions that “nudge” people toward healthier behaviors by modifying the environment to increase the prominence or convenience of target food and drink items have shown promise in increasing the selection of healthy foods from school food service settings [10,11]. A recent systematic review of 29 choice architecture interventions within the school food service setting found that strategies such as point-of-purchase labeling, prompts, and food placement are positively associated with students’ selection of healthier foods [11]. However, there remains little evidence of their longer-term impacts, with 21 of the 29 (72.4%) studies assessing dietary impacts over a period of less than 4 months [11]. Only two studies assessed the intervention impact 12 months or more postbaseline [12,13]. The cluster randomized controlled trial (RCT) by Larson et al [13] found increased participation in a school breakfast program relative to baseline, following the introduction of grab-and-go breakfast carts over a 2-year study, and the nonrandomized trial by Ensaff et al [12] found that an intervention involving repositioning, promoting, and labeling healthy target foods increased the selection of those items measured intermittently over 2 years.

Given that the effects of behavioral interventions typically attenuate over time [14,15], to achieve enduring improvements in public health nutrition, interventions need to be able to support long-term behavior change. Choice architecture interventions may be more resilient to attenuation over time, as they operate mainly through automatic psychological processes, and are not dependent on an ongoing cognitive load or self-regulatory skills of users [16]. However, little is known about their longer-term effects [17]. Although a recent long-term evaluation of the Healthy, Hunger-Free Kids Act of 2010 in US middle schools found that a multicomponent intervention, which included nudges in the form of defaults, found significant increases in the diet quality score, as measured by NHANES up to 4 years later [18], evidence is particularly scant regarding choice architecture interventions delivered online, with only 2 of the 29 (6.9%) studies in the above review delivered online [11] and both with a short follow-up period of only 2 weeks [19] and 2 months [20]. As such, more research is needed to determine the long-term effectiveness of online choice architecture interventions.

Online canteen ordering systems are common in Australian schools and allow parents and students (users) to order and pay for school lunches online. These systems provide unique opportunities to deliver choice architecture strategies to nudge users to select healthier items and assess their long-term impacts. The research team recently conducted the Click & Crunch trial to investigate the impact of choice architecture strategies embedded in an existing online lunch-ordering system in improving the nutritional quality of primary school students’ online lunch order purchases [21]. At 12-month follow-up, the intervention significantly lowered the energy (–69 kJ) and saturated fat (–0.6 g) content of student lunch orders (*P*=.01) without any adverse impact on canteen revenue [21]. Additionally, a higher proportion of healthy or *everyday* items (odds ratio [OR] 1.69; *P*<.001, +9.8%) and a lower proportion of less healthy or *occasional* items (OR 0.68; *P*<.001, –7.7%) were purchased by students at intervention schools compared with controls [21]. While these initial outcomes are promising, an assessment of the longer-term impact of the intervention on student lunch purchases is needed to better quantify its contribution to public health nutrition.

The primary aim of this study was to determine the long-term effectiveness from baseline to 18-month follow-up of the Click & Crunch intervention, a multistrategy behavioral intervention embedded within an existing online lunch-ordering system, in reducing the energy, saturated fat, sugar, and sodium content of primary school students’ lunch orders.

## Methods

### Subjects and Methods

A detailed description of the trial methods is provided in the study protocol [22]. The study was approved and procedures monitored by the Human Research Ethics Committee of the University of Newcastle (reference no. H-2017-0402) and the relevant New South Wales (NSW) Catholic Schools Dioceses (including Sydney, Parramatta, Lismore, Maitland-Newcastle, Bathurst, Canberra-Goulburn, Wagga Wagga, Wollongong, and Wilcannia-Forbes). The trial is reported according to the Consolidated Standards of Reporting Trials (CONSORT) guidelines for cluster RCTs (see Multimedia Appendix 1). The original trial methods and 12-month follow-up were prospectively registered with the Australian New Zealand Clinical Trials Registry (ACTRN12618000855224). The 18-month follow-up was conducted in accordance with previously registered procedures, and all outcomes were registered.

### Design

The trial used a parallel cluster RCT design. Schools with an existing online lunch-ordering system were randomly assigned either to a multistrategy behavioral intervention or a control (standard online ordering system). Outcome data were collected over an 8-week period (weeks 1-8 of the school term) at baseline and again 12 months and 18 months after study initiation. This manuscript reports the 18-month findings. Once the intervention commenced and was switched on within the online ordering system, the intervention strategies remained in place until after collection of the 18-month follow-up data.

### Sample

#### Schools

A sample of 17 (9 intervention, 8 control) nongovernment (Catholic and Independent) schools catering for students aged 5-12 years in NSW, Australia, that were existing users of the Flexischools online school lunch-ordering system were recruited. Schools were ineligible if they (1) had used the system for less than 1 month prior to recruitment, (2) were privately operated (as these externally leased canteens often service multiple schools, increasing the risk of intervention contamination), or (3) catered exclusively for secondary students (due to differences in the NSW Healthy School Canteen Strategy). Combined schools that catered for both primary and secondary students were only eligible to participate if they had a separate menu for primary students (as the NSW Healthy School Canteen Strategy varies by these age groups). Schools were approached to participate by mail and telephone.

#### Users

All students from kindergarten to grade 5 who placed an online lunch order during baseline (term 2, May-July 2018) were included. Students were ineligible if they were in grade 6 or a 5/6 composite class at baseline, as they would have left the school prior to the collection of follow-up data, as were all other nonstudent users of the online ordering system, such as teaching staff and guests.

#### Orders

Only orders placed on a mobile device were included, as users placing orders via a desktop device were not exposed to all intervention strategies. Recurring orders placed prior to intervention commencement were excluded, as were orders placed for food service periods other than lunch (eg, recess or special food days), as users would not have been exposed to all intervention strategies. Given that the system offers the capability for users to purchase items in bulk (eg, for class parties), orders that contained 15 or more items were also excluded. This decision was based on dietitian consensus and knowledge of the number of items that are typically in online lunch orders (eg, on average, a primary school student lunch order contains 2.3 items). Small items, such as chicken nuggets, may be sold individually and then purchased in higher quantities (eg, 6 or 12 nuggets), accounting for the high upper limit for a plausible order.

### Randomization and Blinding

After recruitment and following the collection of the baseline canteen menus, an independent statistician block-randomized schools (block size ranging from 2 to 4) using a random number function in Microsoft Excel to an intervention or a control group in a 1:1 ratio. Randomization was stratified by school sector (Catholic vs Independent) and socioeconomic status (most vs least advantaged) based on the school postcode (Socio-economic Indexes for Areas [SEIFA]) [23].

### Intervention

The intervention is described in full elsewhere [22]. The intervention was guided by principles of choice architecture [16] and used strategies that have been demonstrated to support healthier food choices in similar food service settings [24-29]. Briefly, intervention schools had a series of choice architecture strategies applied to their online menu within the online canteen ordering system. The point at which each intervention school had the strategies switched on varied from August to October 2018, but once switched on, they remained in place for 14-16.5 months until the end of the 18-month follow-up period. The intervention sought to encourage the purchase of healthier foods and beverages aligned to the Australian Dietary Guidelines and NSW Healthy School Canteen Strategy, that is, the intervention encouraged the ordering of food items lower in energy, saturated fat, sugar, or sodium. The intervention strategies included:

- *Menu labeling:* Each menu item was labeled with colored symbols as *everyday*, *occasional*, or *caution* (also known as “should not be sold”) per the NSW Healthy School Canteen Strategy [30], and a key defining the symbols was provided.
- *Positioning:*  *Everyday* menu items and healthier categories (eg, salads and fruit) were positioned most prominently (ie, first), with *caution* and *occasional* items positioned least prominently (ie, middle and last, respectively). *Occasional* and *caution* items with multiple flavors (eg, chips) were displayed on a separate screen that users had to click through to reach.
- *Prompting:* Healthier categories received an attractive image and a text prompt (ie, “This is a good choice.”). Users selecting *occasional* or *caution* hot foods received a prompt to also select fruit, vegetables, or water (“Healthy add-ons”).
- *Feedback:* Prior to users finalizing their orders, they were provided with tailored feedback (ie, a pie graph and accompanying text) based on the proportion of *everyday* items in their order.
- *Incentives:* A reward symbol cartoon character and congratulatory text were printed on the student’s lunch order bag for orders that contained 100% *everyday* items.

#### Canteen Supportive Strategy

In addition, an audit feedback report was emailed to canteen managers and principals at the start of the intervention period, classifying each menu item as *everyday*, *occasional*, or *caution* and providing feedback on substitutable healthier items. It also provided general information about how to price items to encourage healthier purchasing. This targeted the canteen managers rather than parent and student users.

With the exception of the feedback report for canteen managers, all strategies were incorporated directly into the school’s online menu within the online canteen ordering system.

#### Intervention Fidelity

Once per term during the intervention period (ie, approximately every 10 weeks), a research assistant (author RZ) monitored the online menus to record adherence to the intervention strategies. Where unlabeled menu items (eg, new items) were identified, the research team contacted the online lunch-ordering provider to apply the label and intervention strategies accordingly. Given that three of the four other online strategies were programmed based on the label that was applied, correct application of the label ensured these strategies were also correctly implemented.

### Control

The control group did not receive any of the intervention strategies and had access to the standard online ordering system only.

### Data Collection and Outcomes

Purchasing data were automatically collected and stored by the online ordering system and were subsequently extracted for the defined baseline and follow-up data collection periods by Flexischools. The purchasing data for the baseline period were retrospectively collected. Data were collected over three 8-week periods spanning an 18-month period, with baseline occurring during May to July 2018 and the 18-month follow-up occurring during October to December 2019 (baseline: term 2, 2018; 12 months: term 2, 2019; and 18 months: term 4, 2019). The 12-month follow-up was the primary trial endpoint, and 12-month outcomes have been previously published [21]. This paper reports the baseline and 18-month follow-up only in order to examine intervention effectiveness in the longer term.

#### Primary Trial Outcomes

The primary trial outcomes at 18 months were identical to those at 12 months and included the mean total energy (kJ), saturated fat (g), sugar (g), and sodium (mg) content of online lunch orders. These nutrition outcomes were calculated by a dietitian who conducted a comprehensive menu assessment of each school’s canteen menu and applied those values to the online purchasing data that were automatically collected and stored by the Flexischools online canteen ordering system. The dietitian conducted the menu assessment by a telephone interview with each canteen manager and collected the brand, product name, serving size, or recipe for each available item. After the interview, the dietitian generated a nutritional profile for each item. For canteen-made products, the dietitian entered the recipe into FoodWorks nutrition analysis version 9 (Xyris Software) [31]. To assign the nutritional profile (energy, saturated fat, sugar, and sodium content) for prepackaged items, the dietitian consulted a series of sources in the following order: (1) a database of over 2000 commonly stocked canteen products developed by the researchers over the past decade, (2) the FoodFinder database [32], (3) the FoodSwitch website [33], and (4) an internet search for the product’s nutrient panel.

#### Secondary Trial Outcomes

The secondary trial outcomes were as follows:

- *Healthy purchasing outcomes:* The proportion of all online lunch order items that were (1) *everyday*, (2) *occasional*, and (3) *caution*, as classified by the dietitian using the NSW Healthy School Canteen Strategy [30].
- *Revenue (adverse outcome):* Automatically collected purchasing data were used to determine impact of the intervention on the school canteen’s average weekly revenue in Australian dollars.

#### School Characteristics

At baseline, descriptive data regarding school characteristics, such as the number of student enrolments, the proportion of Aboriginal and Torres Strait Islander enrolments, and postcode were collected from a national website (myschool.edu.au) [34].

#### Canteen Characteristics

To describe the sample, data regarding canteen operations (eg, number of days open, model of operation, paid canteen manager) were collected during a canteen manager survey during the initial 12-month follow-up.

### Sample Size Calculation

Sample size estimates were calculated a priori based on a 12-month follow-up and are also described in the trial protocol [22]. The original sample size indicated that a sample of 26 schools was required to ensure a detectable difference of 195 kJ per lunch order, with 80% power, an intraclass correlation coefficient (ICC) of 0.05, and a 0.0125 significance level at 12-month follow-up (Bonferroni-adjusted).

### Statistical Analysis

All outcome data were analyzed under an intention-to-treat approach whereby all student lunch orders and schools were analyzed based on the groups they were originally allocated, and included data from students that had baseline purchasing data.

Primary trial outcomes were assessed using separate linear mixed models by comparing differences between intervention and control groups over time (from baseline to 18 months) through the inclusion of a group-by-time interaction fixed effect. All models included a random intercept for schools (to account for potential school-level clustering), a nested random intercept and random time effect for students (to account for repeat measurements within and over time), and fixed effects for the school sector and SEIFA. All available data were incorporated into the model (baseline, 12 months, and 18 months). The results of the 12-month follow-up are included as a supplementary file (see Multimedia Appendix 2). The unit of analysis for primary trial outcomes was lunch orders, where a lunch order could contain multiple items.

Secondary trial outcomes relating to nutritional quality (*everyday*, *occasional*, *caution*) were assessed using separate logistic mixed models. Changes in the proportion of items ordered belonging to each category (ie, *everyday* items vs *not everyday* items) were compared between intervention and control groups over time by including a group-by-time interaction fixed effect. Similar to primary trial outcomes, all models included a random intercept for schools (to account for potential school-level clustering), a nested random intercept and random time effect for students (to account for repeat measurements), and fixed effects for the school sector and SEIFA and included the 12-month outcomes. Differences in the average weekly revenue were assessed using a linear mixed model with a similar structure to models for the primary outcomes. School and canteen characteristics were previously reported in the 12-month outcome paper and are included here for context.

A per-protocol analysis was also conducted, which included only those schools that had at least 80% of verifiable strategies correctly applied at follow-up. Statistical analyses were performed using SAS version 9.3 (SAS Institute, Cary, NC, USA). As no differences in subgroups (with respect to student grade, school sector, or frequency of canteen use) were found in the 12-month follow-up [21], no analyses were conducted on 18-month data.

## Results

### Sample Characteristics

**Figure 1:**
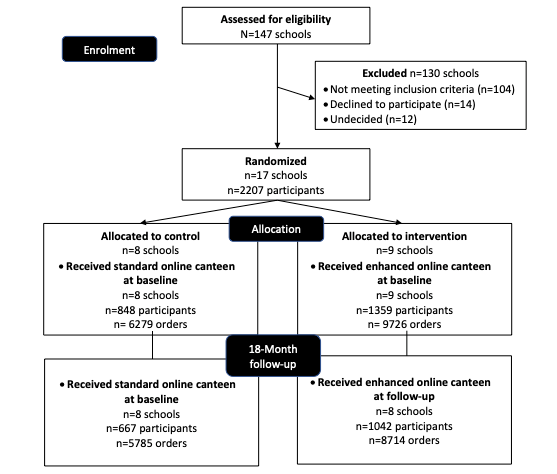
CONSORT diagram. CONSORT: Consolidated Standards of Reporting Trials.

The baseline characteristics of the sample are presented in Tables 1 and 2. Characteristics were similar between groups; however, intervention schools had higher student enrolments and more lunch orders per week than control schools (no significance testing). In total, 1042 intervention students (8 schools) and 667 control students (8 schools) from a total of 16 schools were included in the 18-month follow-up, representing 94% of the 17 schools recruited to the trial (see Figure 1). One intervention school withdrew after the 12-month follow-up, but prior to the 18-month follow-up, and contributed data for two of the three time points in the analysis. The sample also contained two schools with privately operated (externally leased) canteens that did not initially identify as such in the recruitment process. The schools were retained as there was no contamination risk, given that they did not service any other schools in the sample. One was allocated to the control group and one to the intervention group. There were 1435 recurring orders (4.5%) that did not meet the eligibility criteria and were removed from analysis, which resulted in 10 students who only had recurring orders being excluded. Furthermore, four orders were excluded due to being implausibly large based on dietitian assessment.

**Table 1:** School characteristics of the sample at baseline for all participating schools by group [21].

School characteristics reported at baseline			All schools (n=17)		Intervention schools (n=9)		Control schools (n=8)	
**School sector, n (%)**								
	Independent	7 (41.2%)		4 (44.4%)		3 (37.5%)		
	Catholic	10 (58.8%)		5 (55.6%)		5 (62.5%)		
Mean (SD) number of enrolments^a^		443.7 (177.4)		501.3 (207.9)		386 (134.3)		
Mean % of Aboriginal or Torres Strait Islander students		5%		6%		4%		
**Socioeconomic status, n (%)**								
	Least advantaged	7 (41.2%)		3 (33.3%)		4 (50%)		
	Most advantaged	10 (58.8%)		6 (66.7%)		4 (50%)		

^a^Excluding combined schools (as this information was not available on the MySchool website)

**Table 2:** Canteen characteristics of the sample at baseline for all participating schools by group [21].

Characteristics reported at baseline		All schools (n=15)	Intervention schools (n=7)	Control schools (n=8)	
**Type of operation, n (%)**					
	Principal/school run	12 (80%)	6 (85.7%)	6 (75%)	
	P&F^a^/P&C^b^ association run	1 (6.7%)	0 (0%)	1 (12.5%)	
	Contracted food service	2 (13.3%)	1 (14.3%)	1 (12.5%)	
**Type of manager, n (%)**					
	Paid	15 (100%)	7 (100%)	8 (100%)	
	Volunteer	0 (0%)	0 (0%)	0 (0%)	
**Days of operation, n (%)**					
	5 days a week	11 (78.6%)	6 (85.7%)	5 (62.5%)	
	3-4 days a week	3 (20%)	1 (14.3%)	2 (25%)	
	1-2 days a week	1 (6.7%	0 (0%)	1 (12.5%)	
Mean (SD) number of weekly online lunch orders (per school)		—	135.9 (80.3)	98.3 (91.3)	

^a^P&F: Parents and Friends.

^b^P&C: Parents and Citizens.

### Outcomes

In the intervention group, from baseline to 18-month follow-up, online lunch orders contained significantly less energy (–74.1 kJ; 95% CI [–124.7, –23.4]; *P*=.006) and saturated fat (–0.4 g; 95% CI [–0.7, –0.1]; *P*=.003) relative to control orders (see Tables 3-5). There was also a significant between-group difference over time in the odds of lunch orders including *everyday* items (OR 1.2; 95% CI [1.1, 1.4]; *P*=.009) corresponding to a 3.8% increase in *everyday* items purchased among the intervention group, and a significant difference in the odds of lunch orders including *caution* items (OR 0.7; 95% CI [0.6, 0.9]; *P*=.002) corresponding to a 2.6% decrease in *caution* items among the intervention group. There was no between-group difference over time in the sugar (–0.5 g; 95% CI [–1.7, 0.7]; *P*=.4) or sodium (–3.0 mg; 95% CI [–28.0, 22.1]; *P*=.8) content of lunch orders or in the odds of lunch orders including *occasional* items (OR 1.0; 95% CI [0.8, 1.1]; *P*=.6).

**Table 3:** Primary outcomes from baseline to 18-month follow-up.

Primary outcomes	Baseline intervention (n=23,526 items; n=9726 orders; n=1359 children; n=9 schools)	Baseline control (n=14,124 items; n=6279 orders; n=848 children; n=8 schools)	18-month follow-up intervention (n=20,351 items; n=8714 orders; n=1042 children; n=8 schools)	18-month follow-up control (n=12,579 items; n=5785 orders; n=667 children; n=8 schools)
Energy (kJ) mean (SD)	1634.4 (704.2)	1632.1 (743.0)	1603.8 (700.4)	1671.4 (876.1)
Saturated fat (g) mean (SD)	5.2 (3.9)	4.6 (3.2)	4.6 (3.7)	4.3 (3.3)
Sugar (g) mean (SD)	12.9 (14.0)	15.8 (19.1)	13.1 (13.9)	17.3 (24.4)
Sodium (mg) mean (SD)	596.1 (343.0)	599.3 (328.9)	606.1 (409.1)	590.3 (344.3)

**Table 4:** Secondary outcomes from baseline to 18-month follow-up.

Secondary outcomes	Baseline intervention (n=23,526 items; n=9726 orders; n=1359 children; n=9 schools)	Baseline control (n=14,124 items; n=6279 orders; n=848 children; n=8 schools)	18-month follow-up intervention (n=20,351 items; n=8714 orders; n=1042 children; n=8 schools)	18-month follow-up control (n=12,579 items; n=5785 orders; n=667 children; n=8 schools)
% of student lunch order items classified as *everyday*, % (n)	31.6% (7423)	40.4% (5711)	41.5% (8439)	46.6% (5859)
% of student lunch order items classified as *occasional*, % (n)	47.9% (11261)	43.8% (6185)	43.5% (8846)	40.2% (5052)
% of student lunch order items classified as *caution*, % (n)	20.6% (4842)	15.8% (2228)	15.1% (3066)	13.3% (1668)
% of energy from saturated fat, mean (SD)	11.0 (5.9)	9.9 (5.1)	9.8 (6.1)	9.3 (5.1)
% of energy from sugar, mean (SD)	12.0 (11.8)	13.9 (12.7)	12.8 (13.0)	15.7 (15.0)

The per-protocol analysis excluded three of the eight intervention schools that only partially applied the intervention strategies. The pattern of results for the primary outcomes was similar between the per-protocol and main analyses (see Table 5), with significant differences observed for the energy and saturated fat content of lunch orders and slightly larger effect sizes (–93 kJ vs –74.1 kJ). There were no significant differences in the secondary outcomes in the per-protocol analysis at the prespecified Bonferroni-adjusted significance level (*P*=.0125).

**Table 5:** Main vs per-protocol analysis from baseline to 18-month follow-up^a^.

Outcomes		Main analysis differential effect (group by time) (95% CI)	Main analysis OR^b^ (95% CI)	Main analysis *P* value	Per-protocol analysis differential effect (group by time) (95% CI)	Per-protocol analysis OR (95% CI)	Per-protocol analysis *P* value
**Primary outcomes**							
	Energy (kJ)	–74.1 (–124.7, –23.4)	—	0.006	–93.0 (–151.9, –34.2)	—	0.003
	Saturated fat (g)	–0.4 (–0.7, –0.1)	—	0.003	–0.5 (–0.8, –0.2)	—	0.003
	Sugar (g)	–0.5 (–1.7, 0.7)	—	0.39	–0.1 (–1.5, 1.3)	—	0.87
	Sodium (mg)	–3.0 (–28.0, 22.1)	—	0.81	7.3 (–21.9, 36.4)	—	0.61
**Secondary outcomes**							
	% of student lunch order items classified as *everyday*, % (n)	3.8%	1.2 (1.1, 1.4)	0.009	0.6%	1.0 (0.9, 1.1)	0.68
	% of student lunch order items classified as *occasional*, % (n)	–1.1%	1.0 (0.8, 1.1)	0.64	2.6%	1.1 (1.0, 1.3)	0.013
	% of student lunch order items classified as *caution*, % (n)	–2.6%	0.7 (0.6, 0.9)	0.002	–2.6%	0.9 (0.8, 1.0)	0.07
	% of energy from saturated fat	–0.4 (–0.8, –0.02)	—	0.039	–0.6 (–1.1, –0.1)	—	0.02
	% of energy from sugar	–0.08 (–1.0, 0.8)	—	0.86	0.8 (–0.3, 1.8)	—	0.14

^a^All models included a random intercept for school, a nested random intercept and random time effect for students, and fixed effects for sector and SEIFA. All available data was incorporated into the model (baseline, 12-months, 18-months) to describe purchasing patterns over time.

^b^OR: odds ratio.

### Revenue

Analysis of the average weekly canteen revenue indicated that over time from baseline to 18-month follow-up, there were no significant differences between the intervention and control groups ($80.42; 95% CI [–104.48, 265.33]; *P*=.4). This finding was unchanged in the per-protocol analysis (see Table 6).

**Table 6:** Average weekly revenue per school ($^a^).

Baseline intervention, mean (SD)	Baseline control, mean (SD)	18-month follow-up intervention, mean (SD)	18-month follow-up control, mean (SD)	Main analysis differential effect (group by time) (95% CI)	Main analysis *P* value	Per-protocol analysis differential effect (group by time) (95% CI)	Per-protocol analysis *P* value
$668.61 ($420.90)	$496.10 ($442.63)	$1081.03 ($525.54)	$758.76 ($576.13)	$80.42 (–104.48, 265.33)	0.39	$154.56 (–59.15, 368.26)	0.16

^a^All $ amounts are in AUD $. A currency exchange rate of AUD $1=US $0.75 is applicable.

### Quality and Fidelity Checks

Quality checks that were conducted immediately after switching on the intervention identified a technical glitch whereby purchasing a menu item routinely sold in multiples (eg, 6 × chicken nuggets) affected the application of the add-on strategy. The solution was to turn off the strategy for items routinely sold in multiples. Routine fidelity checks conducted once per term during the intervention period did not identify any further technical errors. The fidelity checks indicated that the menu labels were correctly applied to 93%-95% of all online menu items in the first 4 terms of the trial (12 months [21]) and 97% of all items during the last 2 terms (12-18 months). The strategies of positioning, tailored feedback, and incentives were automatically programmed based on the label assigned to each item, and as such, the fidelity checks for the menu labels also applied for these strategies. As previously reported [21], there was a programming issue with the healthy add-on strategies for items sold in multiples (eg, chicken nuggets), and as a result, this strategy was removed only from these items, and two intervention schools in this follow-up had previously requested this strategy be switched off entirely. The presence of the incentive strategy was verified in five of the eight participating intervention schools.

### Availability

The proportion of *everyday*, *occasional*, and *caution* menu items was similar between Independent and Catholic school menus at baseline (59% and 59% *everyday* items, 15% and 16% *occasional* items, and 26% and 25% *caution* items, respectively). Furthermore, the proportion of *everyday*, *occasional*, and *caution* menu items was similar between intervention and control menus at baseline (58% and 61% *everyday* items, 16% and 15% *occasional* items, and 22% and 25% *caution* items, respectively).

## Discussion

### Principal Results

This long-term follow-up of the Click & Crunch intervention using automatically collected purchasing data found significant between-group differences over time from baseline to 18-month follow-up. Specifically, the energy and saturated fat content of intervention lunch orders was significantly lower than controls (–74.1 kJ of energy; –0.4 g of saturated fat). There were no significant between-group differences with respect to sodium or sugar content or the percentage of energy from sugar or fat. Among intervention schools relative to control schools, from baseline to 18-month follow-up, the odds of orders containing *everyday* items were significantly higher (OR 1.2, *P*=.009, corresponding to a 3.8% increase in the purchase of these items), and the odds of orders containing *caution* items were significantly lower (OR 0.7, *P*=.002, corresponding to a 2.6% decrease in the purchase of these items). As such, the results suggest that at 18-month follow-up, the Click & Crunch intervention is effective in reducing the energy and saturated fat content of students’ online lunch orders and in reducing the proportion of unhealthy items and increasing the proportion of healthy items purchased.

### Comparison With Prior Work

The pattern of results was similar between the 12-month and 18-month follow-up. At 12 months (previously reported [21]), the Click & Crunch intervention was effective in reducing the energy and saturated fat content of student lunch orders, and the effects were similar in magnitude to those observed at 18-month follow-up (energy: –69 kJ at 12 months [21] and –74.1 kJ at 18 months; saturated fat: –0.6 g at 12 months [21] and –0.4 g at 18 months). Similarly, there were significant increases in the proportion of *everyday* items purchased among the intervention group (+9.8% at 12 months; +3.8% at 18 months) and significant decreases in the proportion of less healthy items purchased (–7.7% *occasional* items at 12 months; –2.6% *caution* items at 18 months).

There is limited research examining the impact of similar interventions to enable direct comparison of the long-term effects. Multiple systematic reviews of choice architecture interventions to improve dietary outcomes have highlighted the lack of research into long-term intervention outcomes [35,36], including the review of nudge interventions in schools, which reported there are significant knowledge gaps regarding the long-term impact of nudges within the school food environment [12]. However, a nonrandomized longitudinal study investigated the effects of choice architecture strategies (menu labeling and item repositioning) within the physical environment of a hospital cafeteria and recorded sales data from adult employees over a 2-year period [24]. After 2 years, the proportion of healthy items purchased increased by 5% and the proportion of unhealthy items purchased decreased by 4% (*P*<.001) [24], finding changes similar in magnitude to the current study and not providing evidence of label-fatigue over time. Both the school-based studies by Ensaff et al [12] and Larson et al [13] found improvement in the primary outcomes: section of target healthy items and participation in a breakfast program, respectively. Despite study limitations, collectively these findings provide important insights into the durability of choice architecture interventions, suggesting that unlike interventions that require controlled processing, such interventions may be sustained in the long term. As such, they may represent an attractive option for those interested in achieving long-term improvements in public health nutrition via school-based interventions.

Importantly, the magnitude of the effects for energy content and the proportion of *everyday* and *caution* items seen at 18 months appear to have public health significance. The study by Thorndike et al [24] modeled the observed changes in purchasing patterns in a hospital cafeteria 2 years after a choice architecture intervention was implemented and found a 6.2% decrease in total calories purchased and a 4% increase in calories from healthy food purchases [24]. Modeled data on high-frequency cafeteria users suggested that the long-term effects of this intervention could have an impact on obesity rates [24]. However, the change in kilojoules purchased reported in this study and used as the basis for modeling was larger than in the current study. A modeling study based on data from Australian children suggested that a decrease of 100 kcal/day (~418 kJ) would be sufficient to halve the current prevalence of overweight/obesity within a short period (less than 2 years) [37]. As such, the observed long-term decrease in lunch order kilojoule content (–74.1 kJ) is insufficient in isolation to decrease overweight/obesity rates. However, given the relative simplicity of the intervention and the potential for a wide reach, this intervention may play an important role as part of a suite of interventions adopted across multiple settings (school, after-school care and childcare, home, sporting and community clubs, etc) as are commonly adopted by governments in Australia and internationally [38].

### Limitations and Strengths

The limitations of this study are similar to those described in the 12-month outcomes paper [21] and include the relatively small number of schools, the use of purchasing data rather than consumption data, the lack of individual demographic data, and the exclusion of government schools. It also should be acknowledged that the impact of a menu-labeling system on nutritional outcomes is dependent, in part, on the alignment of the labeling system with the target.

Study strengths include a rigorous cluster randomized controlled design, an excellent school retention rate (94%), and a large number of student participants. Furthermore, the evaluation was based on long-term, objective, and real-world purchasing data, which were independently verified, and menu assessments were based on gold-standard processes [39]. Data were also collected at multiple time points, indicating high intervention fidelity.

### Conclusions

To the best of our knowledge, this is the first RCT to investigate the long-term effectiveness of choice architecture strategies applied online. The findings are encouraging and suggest that there are enduring intervention effects up to 18-months postbaseline, including a difference of –74.1 kJ in the energy content and a difference of +3.8% in the proportion of healthy *everyday* items purchased. This provides much needed evidence about the sustainability of multistrategy choice architecture interventions (including menu labeling, positioning, prompting, feedback, and incentives) on children’s school lunch ordering from online canteens. Although the intervention only produced modest effects, given the wide reach of online canteen ordering systems, it may be useful as one of a range of interventions to supplement existing strategies used to improve child diet within the school setting. Further research is required to determine whether the effects transfer to related online food-ordering settings, including groceries, fast food, and meal subscriptions, which are currently used by more than 1.2 billion people worldwide.

## Appendix

Multimedia Appendix 1 CONSORT-eHEALTH checklist (V 1.6.1).

Multimedia Appendix 2 Results from the 12-month follow-up.

## Abbreviations

CONSORT: Consolidated Standards of Reporting Trials
ICC: intraclass correlation coefficient
NHMRC: National Health and Medical Research Council
NSW: New South Wales
OR: odds ratio
P&C: Parents and Citizens
P&F: Parents and Friends
RCT: randomized controlled trial
SEIFA: Socio-economic Indexes for Area
