# Supplementary material for: Long-term Effectiveness of a Multistrategy Behavioral Intervention to Increase the Nutritional Quality of Primary School Students’ Online Lunch Orders: 18-Month Follow-up of the Click & Crunch Cluster Randomized Controlled Trial
Source: J Med Internet Res. 2021 Nov 29;23(11):e31734. doi: 10.2196/31734 (PMC8669584; doi:10.2196/31734)
Supplement: Multimedia Appendix 2 [file jmir_v23i11e31734_app2.docx]

**Supplementary File:**

**Table 1: 12-month outcomes:** Primary and secondary outcomes in intervention and control groups from baseline to 12-month follow-up^a^ (Linear Mixed Model analysis) [20]

| Variable | **Baseline**  **Mean (SD) or % (N)** | | **Follow-up**  **Mean (SD) or % (N)** | | **Intervention vs Control^a^** | | | |
| --- | --- | --- | --- | --- | --- | --- | --- | --- |
|  | **Intervention**  (N=1,359 children; N=9,726 orders; N=23,526 items) | **Control**  (N=848 children; N=6,279 orders; N=14,124 items) | **Intervention**  (N=1108 children; N=9,434 orders; N=22,061 items) | **Control**  (N=691 children; N=6,334 orders; N=14,087 items) | **Main analysis** | | **Per-Protocol analysis** | |
|  |  |  |  |  | **Group by time Differential effect**  (95% CI) | ***P*-value** | **Group by time Differential effect**  (95% CI) | ***P*-value** |
| **PRIMARY OUTCOMES** | | | | | | | | |
| Energy (kJ)^b^ | 1634.4 (704.2) | 1632.1 (743.0) | 1623.3 (699.2) | 1685.6 (838.6) | -69.4  (-119.6, -19.1) | .*010* | -89.4  (-148.9, -29.9) | *.007* |
| Saturated fat (grams)^b^ | 5.2  (3.9) | 4.6  (3.2) | 4.7 (3.7) | 4.9  (3.4) | -0.6  (-0.9, -0.4) | *<.001* | -0.7  (-1.1, -0.4) | *<.001* |
| Sugar (grams)^b^ | 12.9  (14.0) | 15.8  (19.1) | 13.3 (14.5) | 15.4  (21.1) | 0.4  (-0.7, 1.5) | .470 | 0.7  (-0.6, 2.0) | .280 |
| Sodium (milligrams)^b^ | 596.1 (343.0) | 599.3 (328.9) | 580.1 (342.0) | 618.1 (350.7) | -32.1  (-56.3, -7.9) | .013 | -29.9  (-58.1, -1.8) | .039 |
| **SECONDARY OUTCOMES** | | | | | | | | |
| % of energy from saturated fat^b^ (SD) | 11.0% (5.9) | 9.9%  (5.1) | 10.2%  (5.8) | 10.4% (5.2) | -0.9%  (-1.4, -0.5) | *<.001* | -1.1%  (-1.6, -0.5) | *<.001* |
| % of energy from sugar^b^ (SD) | 12.0% (11.8) | 13.9% (12.7) | 12.4%  (11.9) | 13.1% (12.7) | 1.1%  (0.2, 1.9) | .018 | 1.5%  (0.5, 2.5) | *.006* |
| Average weekly revenue per school ($) | $668.60 (420.90) | $496.10 (442.63) | $938.60 (574.07) | $700.81 (480.06) | $65.28  (-76.02, 206.58) | .364 | $119.7  (-20.94, 260.40) | .095 |

*Italics* indicated statistical significance (p<.01)

^a^ Reported values are means & SD unless otherwise indicated. Data were analyzed with the use of separate linear mixed models that were adjusted for SEIFA, and school sector, and clustering at the school and student level.

^b^ Chicken nuggets are commonly sold in multiple units. Some schools pre-package them (i.e. 1 serve = 6 nuggets), whereas other schools allow any quantity to be purchased. To account for this difference, this analysis counted any number of nuggets purchased by a single child at one purchasing occasion to be a single item.

^c^ Baseline ICC values: energy 0.100; saturated fat 0.130; sugar 0.131; sodium 0.111; % of energy from sugar 0.104; % of energy from saturated fat 0.117.

**TABLE 2: 12-month outcomes: Secondary outcomes in intervention and control groups from baseline to follow-up^a^(Logistic Mixed Model analysis)** [20]

|  | **Baseline** | | **Follow-up** | | **Main Analysis** | | **Per-Protocol Analysis** | |
| --- | --- | --- | --- | --- | --- | --- | --- | --- |
| **Items that are:** | **Intervention**  (N=23,526 items) | **Control**  (N=14,124 items) | **Intervention**  (N=22,061 items) | **Control**  (N=14,087 items) | **Relative Odds Ratio**  (95% CI) | ***P*-value** | **Relative Odds Ratio**  (95% CI) | ***P*-value** |
| **‘Everyday’^b^** | 31.55% | 40.43% | 38.61% | 37.45% | 1.7  (1.5, 2.0) | *<.001* | OR=1.5  (1.3, 1.8) | *<.001* |
| **‘Occasional’^b^** | 47.9% | 43.8% | 45.1% | 48.4% | OR=0.7  (0.6, 0.8) | *<.001* | OR=0.7  (0.6, 0.8) | .*001* |
| **‘Caution’^b^** | 20.6% | 15.8% | 16.3% | 14.1% | OR=0.8  (0.7, 1.0) | *.048* | OR=0.9 (0.7, 1.1) | *.390* |

1. separate logistic mixed models were used which included a random intercept for school (to account for potential school level clustering), a nested random intercept and random time effect for students (to account for repeated measurements between and within time points), and fixed effects for sector and SEIFA. Variables were dichotomized e.g. (‘Everyday’ item vs other item).
2. Baseline ICC values: % of ‘Everyday’ foods 0.07; % of ‘Occasional’ foods 0.135; % of ‘Caution’ foods 0.231;
